# Pulmonary Inflammation Is Regulated by the Levels of the Vesicular Acetylcholine Transporter

**DOI:** 10.1371/journal.pone.0120441

**Published:** 2015-03-27

**Authors:** Nathalia M. Pinheiro, Claudia J. C. P. Miranda, Adenir Perini, Niels O. S. Câmara, Soraia K. P. Costa, Maria Isabel C. Alonso-Vale, Luciana C. Caperuto, Iolanda F. L. C. Tibério, Marco Antônio M. Prado, Mílton A. Martins, Vânia F. Prado, Carla M. Prado

**Affiliations:** 1 Department of Medicine, School of Medicine, University of Sao Paulo, São Paulo, Brazil; 2 Department of Biological Science, Federal University of Sao Paulo, Diadema, Brazil; 3 Department of Immunology, Institute of Biomedical Sciences, University of Sao Paulo, São Paulo, Brazil; 4 Department of Pharmacology Institute of Biomedical Sciences, University of Sao Paulo, São Paulo, Brazil; 5 Molecular Medicine Group, Robarts Research Institute, Department of Physiology & Pharmacology and Department of Anatomy & Cell Biology, University of Western Ontario, London, Canada; Chinese Academy of Sciences, CHINA

## Abstract

Acetylcholine (ACh) plays a crucial role in physiological responses of both the central and the peripheral nervous system. Moreover, ACh was described as an anti-inflammatory mediator involved in the suppression of exacerbated innate response and cytokine release in various organs. However, the specific contributions of endogenous release ACh for inflammatory responses in the lung are not well understood. To address this question we have used mice with reduced levels of the vesicular acetylcholine transporter (VAChT), a protein required for ACh storage in secretory vesicles. VAChT deficiency induced airway inflammation with enhanced TNF-α and IL-4 content, but not IL-6, IL-13 and IL-10 quantified by ELISA. Mice with decreased levels of VAChT presented increased collagen and elastic fibers deposition in airway walls which was consistent with an increase in inflammatory cells positive to MMP-9 and TIMP-1 in the lung. *In vivo* lung function evaluation showed airway hyperresponsiveness to methacholine in mutant mice. The expression of nuclear factor-kappa B (p65-NF-kB) in lung of VAChT-deficient mice were higher than in wild-type mice, whereas a decreased expression of janus-kinase 2 (JAK2) was observed in the lung of mutant animals. Our findings show the first evidence that cholinergic deficiency impaired lung function and produce local inflammation. Our data supports the notion that cholinergic system modulates airway inflammation by modulation of JAK2 and NF-kB pathway. We proposed that intact cholinergic pathway is necessary to maintain the lung homeostasis.

## Introduction

Pulmonary diseases such as asthma, acute lung inflammation and chronic obstructive pulmonary disease (COPD) represent major threats to human health. In common, they involve complex immune responses in which inflammatory and epithelial cells release elevated levels of pro-inflammatory Th1/Th2 cytokines such as IL-6, TNF-α, IL-4 and also the regulatory cytokine IL-10 [1]. The persistence of inflammatory processes associated with an imbalance of metalloproteinases (MMP) and their tissue inhibitors (TIMP) induce pulmonary structural changes including fibroblasts/myofibroblasts activation, and extracellular matrix (ECM) fibers deposition [2] which impair lung function.

The cholinergic anti-inflammatory pathway is a modulator of innate immune responses by neuronal and non-neuronal mechanisms [3–6]. This statement is supported by the fact that during the inflammatory process, ACh released by the vagus nerve and acting via α7 nicotinic receptors (α7nAChR) present on macrophage and other immune cells, seems to inhibit cytokine production and thus contributing to counteract an ongoing state of inflammation [6–9]. α7nAChR activation not only inhibits the nuclear translocation of transcription factor NF-kB [10] but it also activates the Janus kinase-2 and signal transducer and activator of transcription 3 pathway (JAK-STAT3). This pathway is involved in the regulation of several cellular functions and is part of the essential chemical signaling to induce cytokine production. However, JAK2-STAT3 can in turn counteracts inflammation by regulating the activity of suppressor of cytokine signaling 3 (SOCS-3) [11].

ACh is synthesized, stored, and released from cholinergic nerve terminals [12, 13] and regulates physiological functions by interaction with two classes of cholinergics receptors, nicotinic and muscarinic [14]. ACh storage in secretory vesicles depends on the activity of the vesicular acetylcholine transporter (VAChT) [15] which is absolutely required for ACh release in the peripheral and central nervous system [16, 17]. Importantly, ACh is also synthesized and released by non-neuronal cells, including immune and epithelial cells [18–22] and the exactly mechanisms involved in non-neuronal ACh release is not fully elucidated. VAChT and other cholinergic components are expressed in non-neuronal cells [12, 19, 20, 23] and at least in cardiomyocytes and in α-cells of the pancreas, the ACh release seems to be VAChT-dependent [12, 24].

In the lung, ACh is known to be released from parasympathetic nerve fibers and to induce bronchoconstriction by biding to muscarinic receptors present in airway smooth muscle and glands [25]. ACh release from lung epithelial cells seems to depend on organic cation transporters (OCT 1 and OCT2) [21, 26]. However, VAChT is expressed in airways and VAChT-positive neurons were found in lung [19, 27]. Rodents with acute lung inflammation showed reduced expression of the cholinergic markers choline acetyltransferase [28], high affinity choline transporter (CHT1) and VAChT in the lung, suggesting a down-regulation of cholinergic activity in asthma physiopathology [19, 21]. Additionally, it has been suggested that the cholinergic system might participate in the pathogenesis of some lung diseases [19] since vagotomy worsened lung inflammation whereas pharmacological stimulation of α7nAChR ameliorate lung inflammation in models of acute lung injury [4, 5]. However, it remains unclear whether lung inflammation is regulated by levels of VAChT.

Here we addressed, using genetic manipulation of VAChT levels [homozygous VAChT knockdown mice (VAChT KD^HOM^) [16], whether release of ACh mediated by this transporter is involved in regulation of lung inflammatory responses as well as lung remodeling and hyperresponsiveness in mice. We also evaluated if VAChT deficiency affected the expression levels of p65-NF-kB and JAK2-STAT3 pathway.

## Methods

VAChT mutant mice were generated as previously described in detail [16]. Heterozygous mice were intercrossed to generate VAChT KD^HOM^ mice and wild-type littermate controls (WT) used in these experiments. These mice were knock-down to VAChT gene and present a reduction in 65% in VAChT levels with correspondent decrease in ACh release which causes myasthenia, cognitive deficits and cardiac dysfunction [15, 17, 29, 30]. The activity of choline acetyltransferase was not modified in these mice [16]. All mice used in this study received humane care in compliance with the “Guide for Care and Use of Laboratory Animals” (NIH publication 85–23, revised 1985).The animals were kept on a 12 h light/dark cycle in a temperature-controlled room at 21–23°C, with free access to water and food. All experiments described in this study were approved by the Internal Ethical Committee of Faculty of Medicine of the University of São Paulo (São Paulo, Brazil) (Document number 0766/08).

### Experimental groups

Male mice (6–8 weeks) of the correct genotypes were randomly assigned to two groups: a. mutant homozygous animals (VAChT KD^HOM^), b. wild-type animals (WT).

### Wire-Hang test

To evaluate the motor function and confirm phenotype of mutant mice, the wire-hang test was performed. Mice were placed on cage top lid and then were slowly inverted for a maximum time of 60 s. The time upside down was measured [31].

### Pulmonary mechanics evaluation

Animals were anesthetized with thiopental sodium (50 mg.kg^-1^, i.p.), tracheostomized and connected to a ventilator for small animals (FlexiVent, SCIREQ, Montreal, Canada). Animals were ventilated at 150 breaths/min with a tidal volume of 10 mL.kg^-1^. The jugular vein was dissected and a polyethylene tubing (Intramedic, Batavia, IL) was tied to infuse different doses of methacholine (10–3,000 μg/kg). Methacholine was infused each 2 minutes and the data were recorded at 30 sec after the end of infusion. The respiratory system resistance (Rrs) and elastance (Ers) were measured according to the linear equation of motion of the respiratory system, as previously described [32] in baseline and after each dose. We analyzed each value and the percentage of maximal response related to baseline.

### Bronchoalveolar Lavage Fluid (BALF)

At the end of the mechanical evaluation, the anterior chest wall was opened, animals were exsanguinated via the abdominal aorta and the BALF was collected as previously described [33]. The trachea was cannulated and BALF was obtained by washing the airway lumina with 3 x 0.5 mL of sterile saline. The recovery volume was over 95% of the instilled fluid and was put into a test tube on ice. To perform total and differential cell counting, the BALF was centrifuged at 800x for 10 min and the cell pellet was ressuspended in 0.2 ml of sterile saline. The total number of viable cells was determined in a Neubauer hemocytometer counting chamber. Differential cell counts were performed in cytocentrifuge preparations of BALF (450 rpm for 6min) (Cytospin, Cheshire, UK) stained with Diff-Quick (Biochemical Sciences Inc., Swedesboro, NJ). At least 300 cells were counted according to standard morphologic criteria.

The amount of total protein in BALF was assayed using BradFord’s method (Protein Assay, Bio-rad, California, USA) with bovine serum albumin (BSA, Sigma-Aldrich, Missouri, EUA) as a standard curve. Absorbance was read at 595 nm (Epoch—Bioteck, Vermont, EUA) and protein levels in mg/mL of BALF were calculated.

### Lung Morphometry

After BALF collect, lungs were infused with 2mL of formaldehyde and were removed *en bloc*. The lungs were fixed with 4% formaldehyde for 24 h and then transferred to 70% ethanol prior to paraffin embedding. Five-micrometer thick sections from embedded paraffin lungs were stained and submitted to histopathological analysis [34].

### Peribronchovascular Edema and Inflammatory Cells

We evaluated the edema area and airway inflammatory cells around the airway using an integrating eyepiece with a known area (10^4^ μm^2^ of total area) in H&E stain section by point-counting technique [34]. To determine the area of edema we counted the number of points of the integrating eyepiece falling on areas of peribronchovascular edema in three to four areas of each airway wall (3–5 airways per animal, 20 fields per animal). The results were showed as edema/area. To determine the polymorphonuclear (PMN) and mononuclear cells (MN) around the airway (between the bronchial epithelium and the adventitia), we counted the number of points of the integrating eyepiece falling on areas of peribronchial inflammation in three to four areas of three to five airway wall and the number of cells in this same area (20 fields per animal). The results were expressed by cell/area. All analyses were performed in randomly selected transversely sectioned airways at a magnification of 1,000x.

### Extracelular Matrix Fibers Deposition

Histological sections were stained for collagen fibers using Sirius-Red (Direct Red 80, C.I. 35780, Aldrich, USA) and for elastic fibers using Oxidate Weigert’s Resorcin-Fuchsin. Using a Leica DM4000B microscope (Leica Microsystems, Wetzlar, Germany), a digital camera (Leica DFC420 Leica Microsystems) and the image analyses software Image Proplus 4.5 (Media Cybernetics, Bethesda, USA), we measured collagen and elastic fibers deposition in the area compressed between epithelial basal membranes until airway adventitia. Five airways at 400x magnification were evaluated for each animal. The positive area of collagen and elastic fibers were expressed as a percentage of the total airway wall area [34].

### Immunohistochemistry evaluation

Immunohistochemical staining was performed using anti-p-65-NF-kB (1:300), anti-MMP-9 (1:600) and anti-TIMP-1 (1:100) antibodies (Santa Cruz Biotechnology, Santa Cruz, CA), by the biotin–streptavidin–peroxidase method. Using the same point counting technique described above, we assessed cells positive for p65-NF-kB, MMP-9 and TIMP-1. Counting was performed in 20 fields of airway wall samples for each animal (5 airways per animal) at 1,000 magnification. Results were expressed as positive cells per area (10^4^μm^2^) [34, 35]. All morphometric analysis was performed by two researchers blind to the genotype.

### Cytokine Measurements

Lung were removed and quickly frozen to perform cytokine measurements by ELISA. We test samples for the presence of TNF-α, IL-6, IL-4, IL-13 e IL-10 in lung homogenate using commercially available kits and according to instructions supplied by the manufacturer (R&D Systems, Minneapolis, MN, USA). All cytokines in lung homogenate were expressed in pg of cytokines/mg of total protein.

### Western Blot Analysis

Western blot was performed using the protocol modified from [36]. Fragments of lung containing approximately 20 μg of spinal cord or lung tissue were homogenized in a boiling extraction buffer [10% SDS, 100 mM Tris (pH 7.4), 10 mM EDTA, 10 mM sodium pyrophosphate, 100 mM sodium fluoride, 10 mM sodium vanadate] with a Polytron PTA 20S generator (model PT 10/35, Brinkmann Instruments, Inc., Westbury, NY) operated at maximum speed for 30 sec. The extracts were centrifuged at 15,000xg, 4° C, for 40 min to remove insoluble material. Protein concentrations of the supernatants were determined by the Bradford assay and an equal amount of total protein from each sample (75 μg) was treated with Laemmli buffer containing dithiothreitol 100 mM. Samples were heated in water bath for 5 min, after which they were subjected to SDS-PAGE (10% bis-acrylamide). Electrotransfer of proteins from gel to nitrocellulose membrane was performed for 90 min at 15V (constant). Nonspecific protein binding to nitrocellulose was reduced by pre incubating the membrane overnight at 4° C in blocking buffer (2.5% milk/TBST). Antibodies used for immunoblot were: anti-VAChT (Abcam, Cambridge, Massachusetts) (1:1,000), anti-M2 mAChR (1:10,000), anti- α7 nAChR (1:500), anti-CHT1 (1:1,000), anti-AChE (1:1,000) (Abcam, Cambridge, Massachusetts), anti-JAK-2 (1:1,000) anti-SOCS-3 (1:1,000), anti-STAT-3 (1:1,000), anti-p65-NF-kB (1:200) (Santa Cruz Biotechnology, Santa Cruz, CA), anti-phosphorylate STAT-3 (1:1,000) (Cells Signaling, Danver, Massachusetts) and anti-β-actin (1:1,000) (Sigma Aldrich, St. Louis, MO) diluted in blocking buffer overnight at 4° C. The membranes were then washed for 30 min with TBST. Bound antibodies were detected with horseradish peroxidade-conjugated (HRP-conjugated) anti-IgG (1:10,000) and visualized by chemiluminescence using UVItec (UVItec Limited, Cambridge, Massachusetts). Band intensities were quantified using UVItec Image Program. The CHT1 and α7 nAChR, the JAK-2, SOCS-3 and p-65-NF-κB and the STAT3 and p-STAT3 were performed in the same gels, respectively, and for this reason the representative β-actin for each of these group was the same.

### RNA extraction, reverse transcription and quantitative real-time PCR (Real-Time qRT-PCR)

Total RNA from lung or spinal cord was extracted with Trizol (Invitrogen Life Technologies, Carslbad, CA), analyzed for quality on agarose gel and absorbance ratios of 260/280 and 260/230 nm, and reverse transcribed to cDNA using the SuperScript III cDNA kit (Invitrogen Life Technologies). Gene expression was evaluated by real-time qRT-PCR using a Rotor Gene (Qiagen, Roermond, Netherlands) and SYBR Green as fluorescent dye (Qiagen) with GAPDH as a housekeeping gene. The reaction conditions were as follows: 95°C for 5 minutes, then 40 cycles of 95°C for 5 seconds and 60°C for 10 seconds. PCR products were run on agarose gel to confirm the size of the fragment and specificity of amplification. Primers used and annealing temperatures are presented: GAPDH (5’-3’ sense: CCACCACCCTGTTGCTGTAG; 5’-3’antisense: CTTGGGCTACACTGAGGACC; 60°C; NM_008084) and VAChT (5’-3’ sense: CCCTTTTGATGGCTGTG; 5’-3’ antisense: GGGCTAGGGTACTCATTAGA; 60°C; NM_10167164). Data were obtained as ct values (ct = cycle number at which logarithmic PCR plots cross a calculated threshold line) and used to determine Δct values (Δct = (ct of the target gene)—(ct of the housekeeping gene). Data were expressed as arbitrary units using the following transformation [expression = 1000x(2^-Δct^) arbitrary units (AU)].

### Statistical Analysis

Statistical analysis was performed using SigmaStat software (SPSS Inc., California, USA). All data were expressed as mean ± SEM. T-test student was performed between groups. The significance level was adjusted to 5%.

## Results

### VAChT levels are reduced in lung and spinal cord

**Fig 1:**
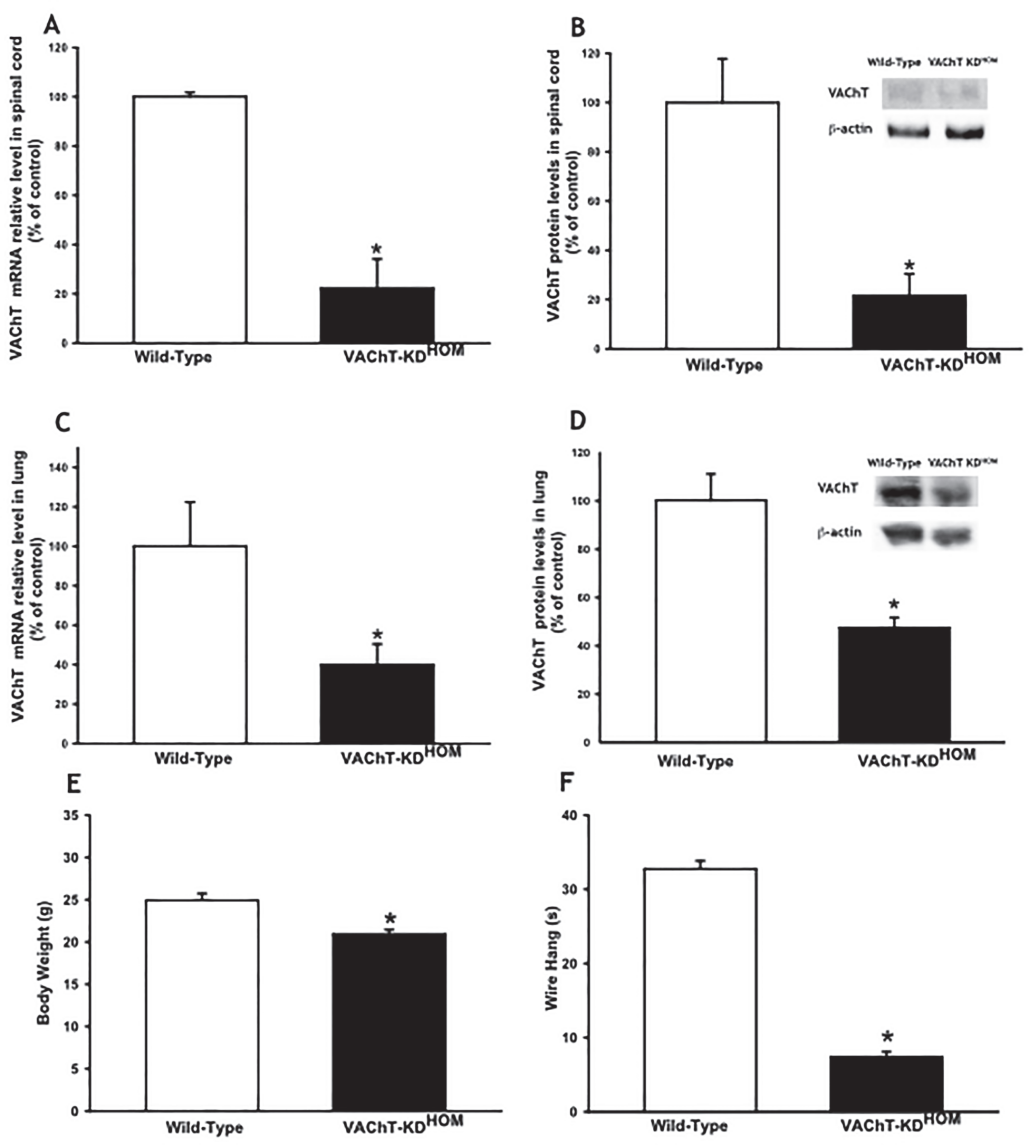
Protein content and mRNA levels of VAChT. A and B. VAChT mRNA expression (A) measured by real-time PCR (6–8 mice per group) and protein content (B) quantified by Western Blot (3 mice per group) in spinal cord from wild-type and VAChT-KD^HOM^ (mutant). **C and D**. VAChT mRNA expression (C) measured by real-time PCR (6–8 per group) and protein content (D) quantified by Western blot (6–8 mice per group) in lung from wild-type and VAChT-KD^HOM^ (mutant). Glyceraldehyde 3-phosphate dehydrogenase (GAPDH) was used as a housekeeping for gene expression analysis (A and C) and β-actin was used as protein loading control for Western Blot (B and D). Both were presented as a percentage of WT. The gels (B and D) is representative of results that were obtained in an experiment that was repeated two times. *p<0.05 vs wild-type mice. **E and F:** Body weight in g (E) in wild-type and mutant mice and the time in wire hang test performed before all measurements in seconds (F). E and F represent data of 8–14 animals per group. Data area expressed as means ± SEM. *P<0.001 vs. wild-type mice.

In order to confirm the phenotype of mutant mice, we quantified VAChT mRNA and protein level in spinal cord (1A and 1B) or in lungs (1C and 1D). As expected we found approximately 80 and 60% of the wild-type VAChT mRNA and protein content in spinal cord and in the lung of VAChT mutant mice, respectively (Fig. 1A-D) (p<0.05). These mutant mice also presented reduction in body weight and in the time spent upside down in the wire hang test (Fig. 1 E-F, respectively) (p<0.001).

### VAChT-deficiency did not affect AChE, M2, CHT1 and α7nAChR in lung homogenate

**Fig 2:**
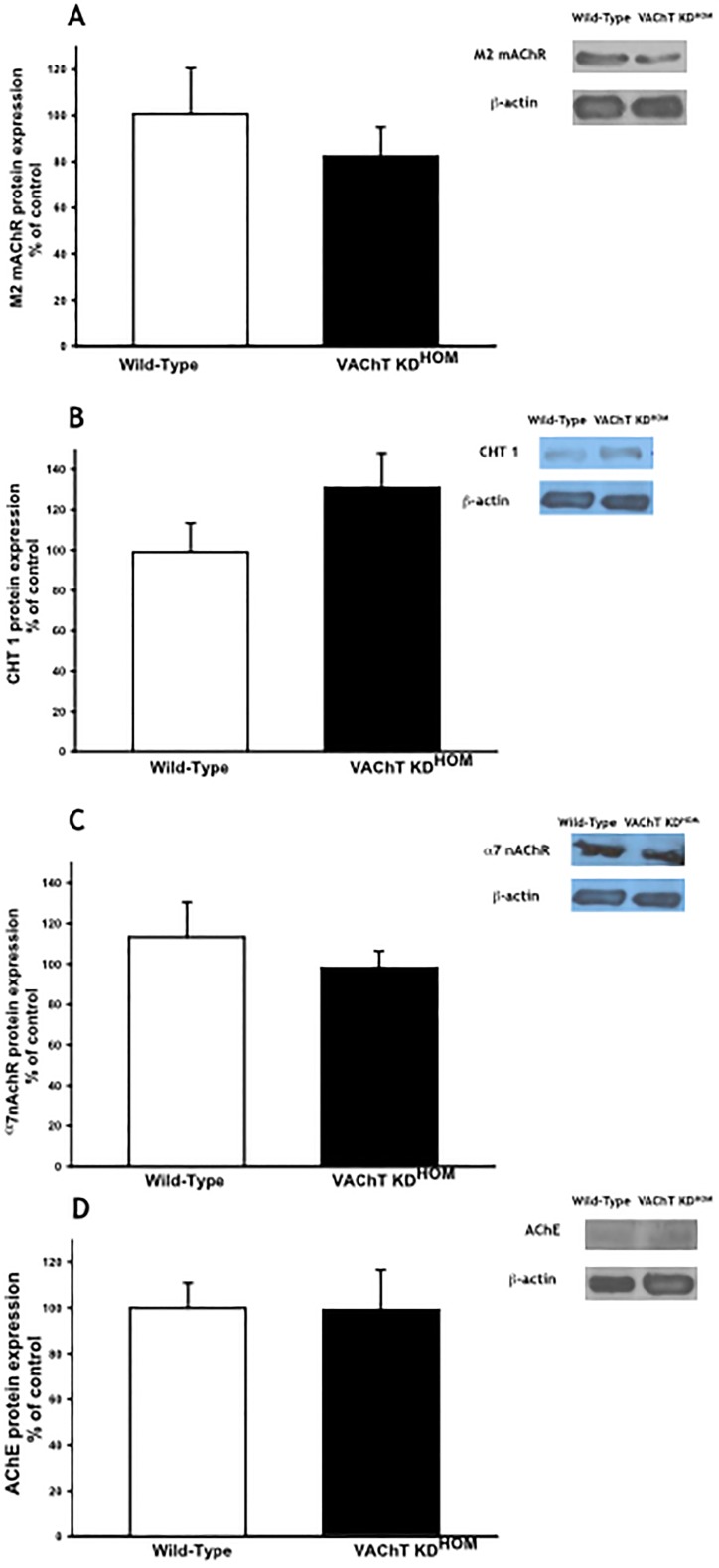
VAChT deficiency did not affect other cholinergic component in lung. Muscarinic receptor 2 (M2), high-affinity choline transporter (CHT1), α7 nicotinic acetylcholine receptor (α7nAChR) and Acetylcholinesterase protein expression was analyzed by Western Blot. The gel is representative of results that were obtained in an experiment that was repeated two times. The graphs represent the values normalized by β-actin.

In order to understand if the reduction of VAChT induced any modification in other cholinergic compounds in lung, we quantified protein content of M2, CHT-1, α7nAChR and AChE. We did not find any significantly difference between the groups (Fig. 2A to D, respectively).

### VAChT-deficiency increases leucocytes in lung

**Fig 3:**
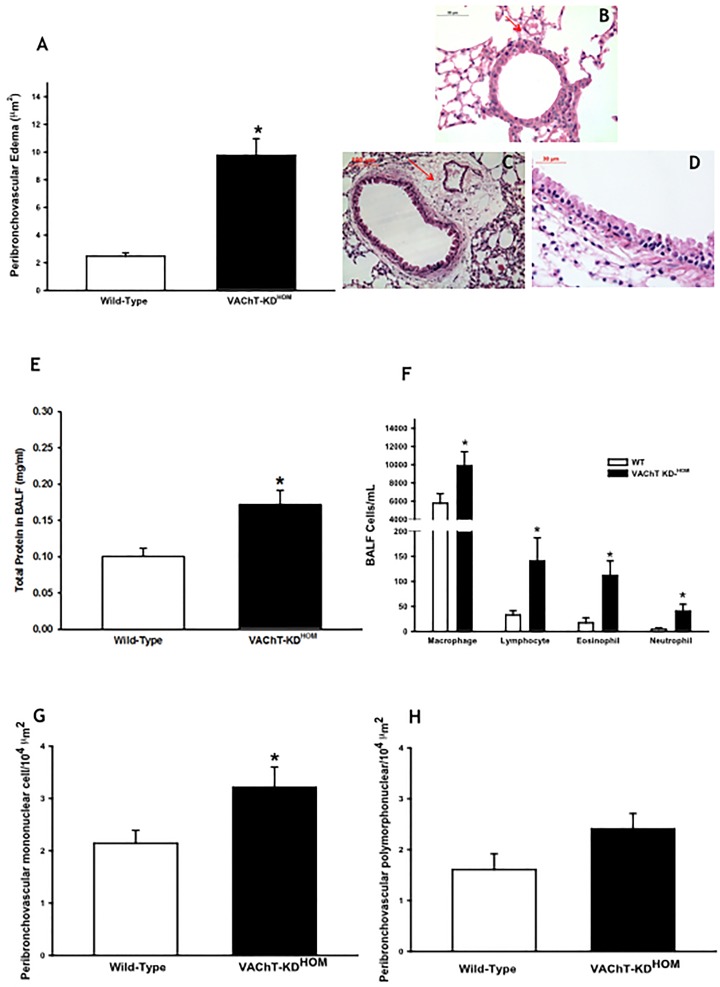
VAChT deficiency increased peribronchial edema and pulmonary inflammation. A. Peribronchial edema evaluated around airways. Lung were fixed in 10% formalin and embedded in paraffin before sections were cut and stained with hematoxylyn and eosin. VAChT KD-^HOM^ increased peribronchovascular edema (*p<0.001 *vs* wild-type mice). B to D. Representative photomicrographs illustrating the peribronchial edema and cellular infiltration around airways obtained from a VAChT mutant mice (C and D) compared to wild-type (B). E. Amount of total protein measured in bronchoalveolar lavage (BALF) (n = 7–8 per group, *p<0.01 *vs* wild-type mice). F. Mean and standard error of macrophages, lymphocytes, eosinophils and neutrophils counted in bronchial alveolar lavage fluid (BALF) (n = 7–8 per group, *P<0.05 *vs* wild-type mice). G and H represent peribronchovascular mononuclear (*p<0.05 *vs* wild-type mice) and polymorphonuclear cells, respectively, evaluated around airways.

In order to examine the possibility that VAChT deficiency affects lung inflammatory responses, we evaluated plasma extravasation edema, one of the primary signals of inflammation due to increase vascular permeability. We found that VAChT deficiency induced an intense peribronchovascular edema (Fig. 3A) in mutant mice compared to WT (p<0.001). Representative photomicrographs of lung slices stained with H&E are shown in panels 3B, C and D. Airways obtained from WT group showed scarce peribronchial infiltrate (Fig. 3B) whereas VAChT KD^HOM^ lung slices presented peribronchovascular edema with inflammatory cells (Fig. 3C and D).

We also evaluated the amount of total protein in BALF, an indirect measurement of lung edema. The mutant mice showed increased levels of total protein in BALF compared to WT animals (p<0.01, Fig. 3E).

We then investigated cellular infiltration in both BALF (Fig. 3F) and in airway wall (3G and H). In agreement with the edema data, mutant mice had increased macrophages, lymphocytes, eosinophils and neutrophils recovered in the BALF (F) compared to WT (p<0.05). In airways, we found an increase in mononuclear cells (G) around airways compared to WT (p<0.05). Although there seems to be a tendency for an increase in the number of polymorphonuclear cells (H) in airways in mutant mice, this was no statically significant.

### VAChT-deficiency increases TNF-α and IL-4 in lung tissue

**Fig 4:**
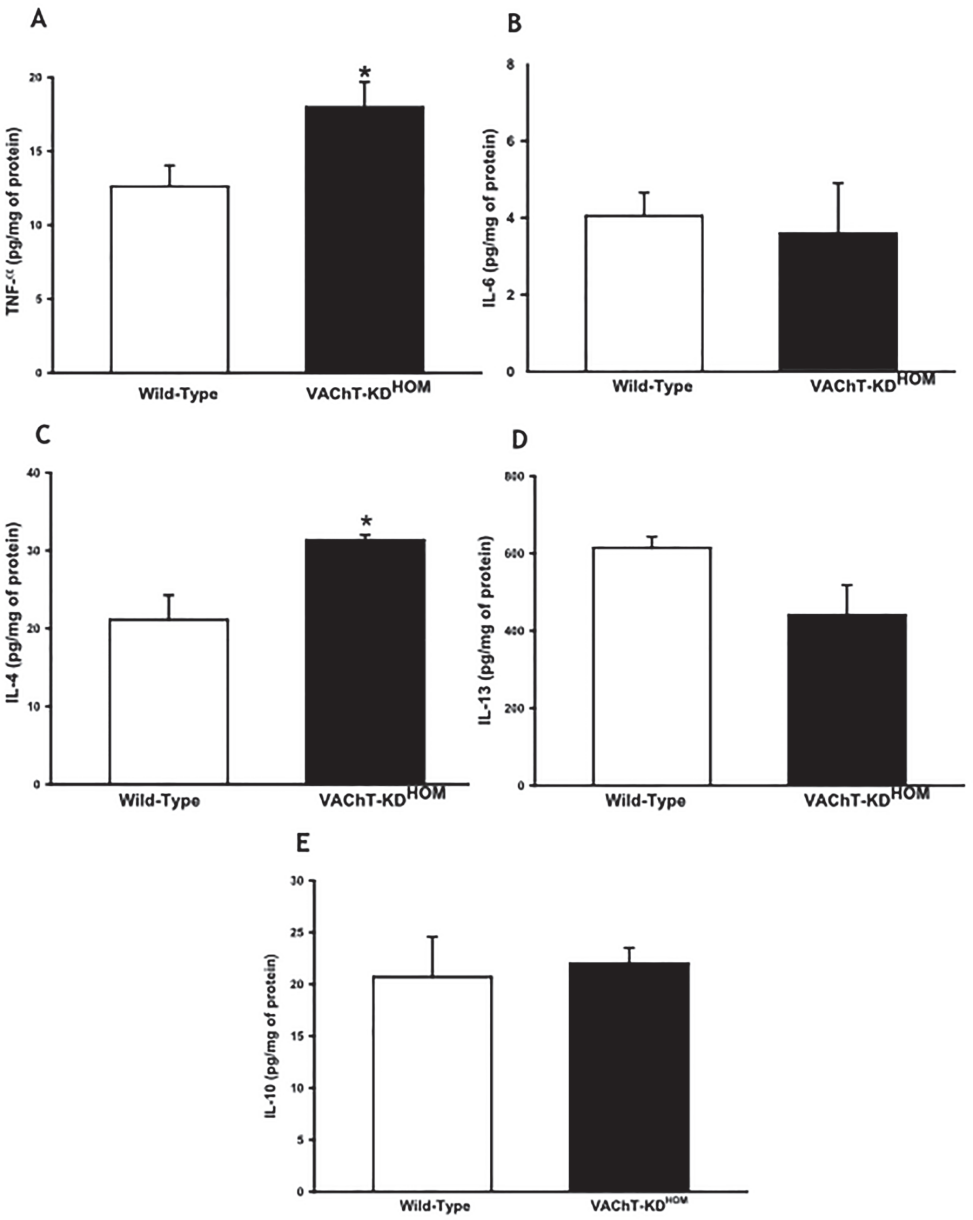
VAChT-deficient mice presented high levels of pro-inflammatory cytokines. Data are expressed as mean ±SEM of five to eight mice per group. Cytokines was measured by ELISA in lung homogenate. Mutant mice (VAChT KD-^HOM^) presented high values of TNF-α and IL-4 compared to wild-type animals. *p<0.05 *vs* wild-type mice.

To further investigate inflammatory responses in cholinergic deficient mice in the absence of any insult we measured pro-inflammatory (TNF-α, IL-6, IL-4, and IL-13) and regulatory cytokine IL-10 (Fig. 4A to E, respectively). Interestingly, TNF-α and IL-4 values were higher in VAChT KD-^HOM^ animals compared to control wild-type (p<0.05). However, the lung content of IL-6, IL-13 and IL-10 was similar between WT and mutant mice.

### VAChT-deficiency modulates pulmonary subunit p65-NF-kB and JAK-2-STAT-3 pathways

NF-kB is a pro-inflammatory nuclear factor and the p-65 subunit plays a crucial role in inflammatory and immune responses and recent evidence suggests that the cholinergic system controls inflammation by inhibiting the NF-kB activation through stimulation of α7-nACHR [8] and activation of JAK-STAT pathway [37].

**Fig 5:**
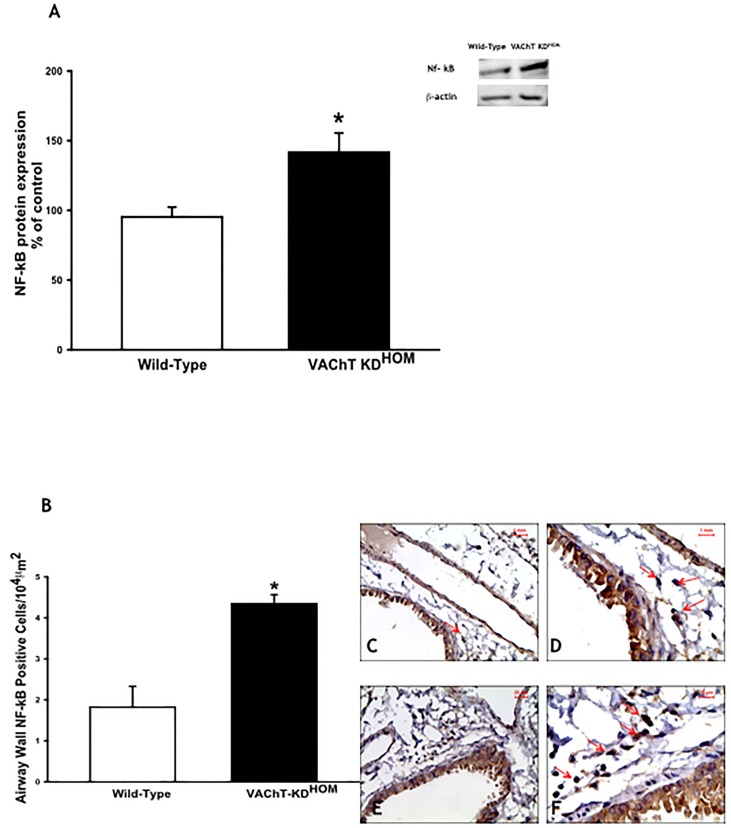
VAChT deficiency increased p65-NF-kB expression in lung. Subunit p65-NF-kB protein expression was measured by western blot (A). The gel is representative of results that were obtained in an experiment that was repeated two times. The graphs represent the values normalized by β-actin (n = 5 per group). *p<0.05 *vs* wild-type group. Number of inflammatory cells positive to p65-NF-kB (B) from 6–8 animals per group was visualized by immunohistochemistry in paraffin embedded section. Representative photomicrographs used to detect NF-kB (Panels C to F) showed a stronger stain in mutant mice (E and F) compared to wild-type mice (C and D). Arrows indicate positive cells around airway wall. *p<0.001 *vs* wild-type group.

We evaluated the protein expression of p-65-NF-kB in lung homogenate. We found an increase in lung expression of NF-kB in mutant mice compared to WT group (p<0.05) (Fig. 5A). In order to observe if the airway wall cells expressed p-65-NF-kB, we also quantified the NF-kB positive cells stained by immunohistochemistry around airways wall (Fig. 5B) by morphometry. VAChT KD^HOM^ mice had increased number of NF-kB-positive cells around airways compared to WT (p<0.001). All together, these data suggest an activation of this pathway in cholinergic deficient mice. In photomicrographs from wild-type (C and D) and VAChT mutant mice (E and F), we can observe positive and negative cells to NF-kB around airways. An increase in the number of cells positive to NF-kB is observed in airway wall obtained from mutant mice (E and F).

**Fig 6:**
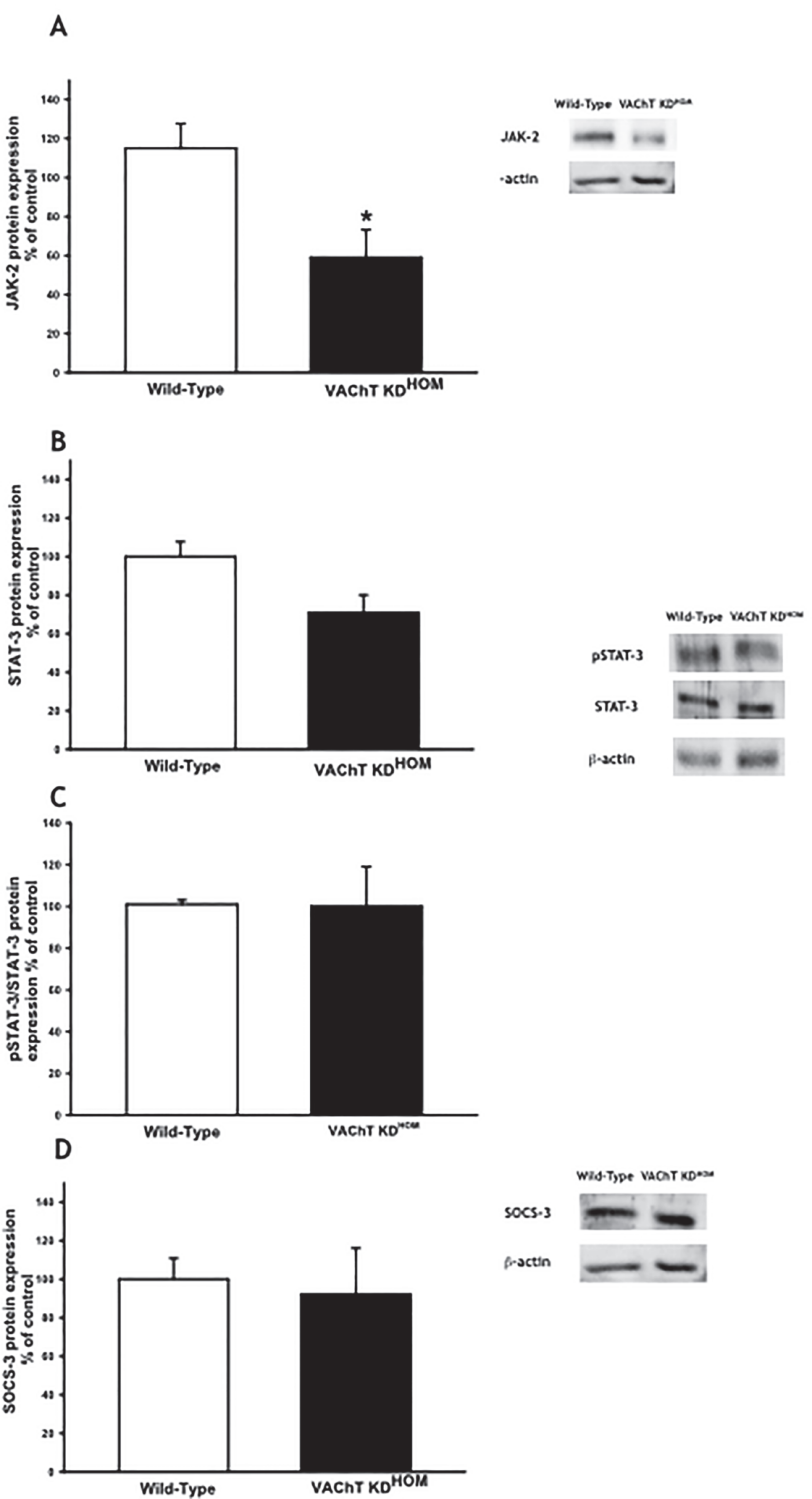
VAChT deficiency reduced JAK-2 expression in lung. Janus kinase 2 (JAK-2) (A), signal transducer and activator of transcription 3 (STAT3) (B) and phosphorylated STAT3 (C) and suppressor of cytokine signaling 3 (SOCS-3) (D) protein expression was measured by Western Blot. The gel is representative of results that were obtained in an experiment that was repeated two times. The graphs represent the values normalized by β-actin. *p<0.05 *vs* wild-type group.

We also evaluated in lung homogenate the protein expression of JAK-2, STAT-3, pSTAT-3 and SOCS-3. We found a reduction in lung expression of JAK-2 in mutant mice compared to WT group (p<0.05) (Fig. 6A). There was no significantly difference in STAT-3, pSTAT-3 and SOCS-3 content (Fig. 6B, 6C and 6D). Although a tendency in a reduction of total STAT-3 expression could be observed, the ratio of p-STAT3/STAT3 was not different between the groups, suggesting that STAT-3 was not differently activated in this model.

### Effects of VAChT deficiency in airway ECM fibers deposition

**Fig 7:**
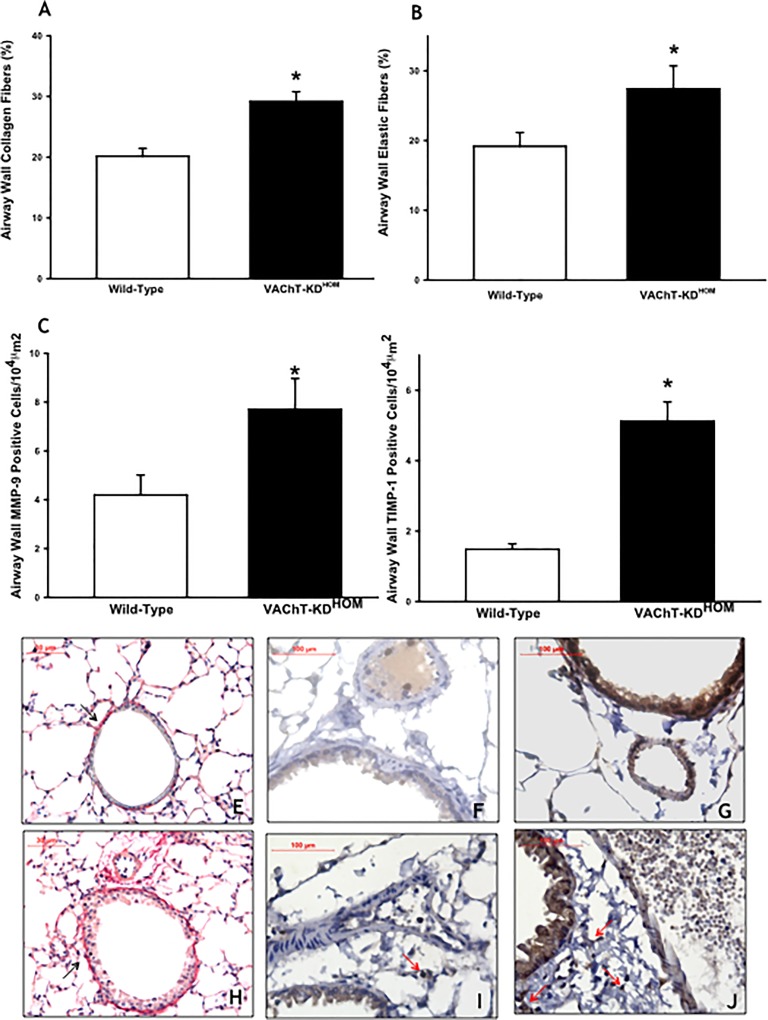
VAChT-deficient induced airway remodeling. Data of collagen (A) and elastic fibers content (B) are expressed as mean ±SEM at twelve to fourteen mice per group. It was evaluated in paraffin sections stained with Picro-Sirius and Resorcin-Fuchsin respectively, and it was measured around airways using an image analysis system. Number of inflammatory cells positive to MMP-9 (C) and TIMP-1 (D) from 6–8 animals per group was visualized by immunohistochemistry in paraffin embedded section. Collagen content was enhanced by VAChT deficiency and it can be observed in panels E (wild-type) and H (mutant mice). Representative photomicrographs used to detect MMP-9 (Panels F and I) and TIMP-1 (panels G and J) showed a stronger stain in mutant mice. A and D *p<0.001 *vs* wild-type group and B and C *p<0.05 *vs* wild-type group.

It is well-know that airway remodeling is an important feature of lung disease, and it is usually associated to chronic inflammation [2]. Given that VAChT mutant mice appear to have higher inflammatory response even in the absence of any injury, we asked whether remodeling of airways was also affected. In Fig. 7A and 7B, we found that VAChT deficiency induced an increase in both collagen and elastic fiber content around airways compared to WT group (p<0.001 and p<0.05, respectively).

An imbalance in MMP-9 and TIMP-1 expression level is involved in ECM matrix deposition [38]. Therefore, we used immunohistochemistry analysis to investigate the cells positive for MMP-9 and TIMP-1 expression in airways. Mutant mice (VAChT KD^HOM^) showed increased number of MMP-9 (7C) and TIMP-1 (7D) positive cells when compared to WT, p<0.05 and p<0.001 respectively]. MMP-9 was less than twice greater in VAChT KD^HOM^, whereas the increase in TIMP-1 was approximately three times higher than observed in WT group, tipping in favor of decrease proteolysis supporting the turn-over of the extracellular matrix fibers and the remodeling [38].

Representative photomicrographs stained with Picro-Sirius to detect collagen fibers were shown in Fig. 7E and 7H. We noted that airways obtained from WT group showed a weak staining for collagen fibers around the airway wall in the tissue section (Fig. 7E), coincident with the maintenance of the histoarchitecture of the ECM (arrows). In contrast, lung slices obtained from mutant mice presented increased collagen fiber deposition around airways (Fig. 7H). Panels F, I, G, and J showed positive cells to MMP-9 and TIMP-1, respectively in the two groups studied. VAChT-KD^HOM^ presented an increase in positive cells for MMP-9 and TIMP-1 (arrows) around airways compared to WT.

### VAChT-deficiency increases airway resistance and elastance

**Fig 8:**
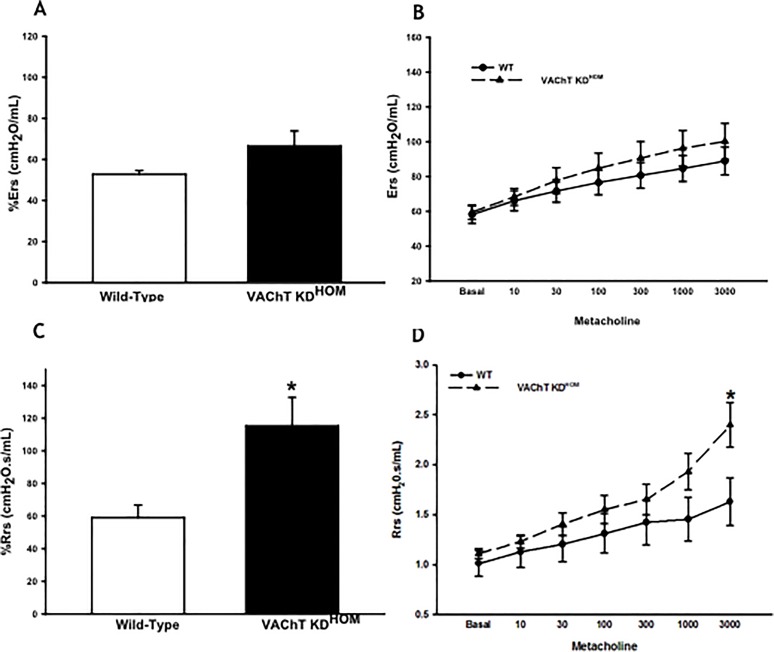
Airway hyperresponsiveness in VAChT deficiency animals. Respiratory system elastance (Ers) (A and B) and resistance (Rrs) (C and D) was recorded in wild-type and mutant mice. We performed a dose response curve to methacholine and values were obtained 30 seconds after each infusion (panels B and D). We also analyzed the percentage of maximal responses related to baseline (A and C). Data are expressed as mean and standard error of the 6–8 animals per group. *p<0.05 compared to wild-type.

Given the increase in inflammation in VAChT deficient mice, we then evaluated both Respiratory system resistance (Rrs) and elastance (Ers) at baseline and post-challenge (after dose-response curve to methacholine) in wild-type and mutant mice. The absolute values of Rrs and Ers obtained in the methacholine dose-response curve are shown in Fig. 8A and 8B, respectively. Baseline values were not significantly different between the groups (8A and B). To further evaluate differences in the airway hyperresponsiveness, an important feature of some respiratory diseases such as asthma, we compared the maximal percentage of Rrs and Ers (C and D), and we found that mutant mice had an increase in the percentage of maximal response of Rrs (8C) compared to wild-type (p<0.05), which could be evidenced in the Rrs response to maximal dose of methacholine (p<0.05).

## Discussion

The major finding of the present study was that VAChT deficiency induces a pro-inflammatory *milieu* in the lung. These effects were associated with an increase in infiltration of inflammatory cells edema and increased in the number of cells expressing NF-kB and a reduction in JAK-2 levels in lung. These results suggest that long-term cholinergic deficiency affects pulmonary inflammation, pointing out the importance of acetylcholine in control pulmonary homeostasis.

Known sources of ACh for the lung are the parasympathetic neurons which are dependent on VAChT to release ACh [17, 30], airway epithelial cells [19, 20], and immune cells [18, 21, 39], in which the dependence of VAChT was not completely understood [26]. In airways, ACh release from parasympathetic nerves is a well-recognized bronchoconstrictor and for this reason anti-muscarinic drugs are recommended to asthmatics and COPD patients. A role for the cholinergic anti-inflammatory system has been described in models of acute systemic inflammation [10, 40, 41]. The cholinergic anti-inflammatory system seems to depend on vagus nerve stimulation and on additional non-neuronal cholinergic source, such as a population of lymphocytes in the spleen [3]. These lymphocytes release ACh that acts as an autocrine and a paracrine mediator of cytokine release from macrophages [3, 7, 18]. Furthermore the stimulation of α7nAChR ameliorates lung inflammation in a model of acute lung injury [4, 5]. However, it is unknown whether VAChT and endogenous ACh is involved in the maintenance of lung homeostasis.

In order to evaluate the effects of cholinergic reduction in lung, we used genetically modified mice with cholinergic dysfunction. These mice were produced by targeting the VAChT gene. The release of ACh in these animals is proportional to the levels of VAChT expression [16, 30] and VAChT KD^HOM^ mice have approximately 65% reduction in the levels of VAChT in the whole body. Here, we checked the VAChT mRNA expression in spinal cord and lung and confirmed this reduction, that was around 80 and 60%, respectively. These data were also confirmed by the reduction in VAChT protein content both in lung and in spinal cord. Furthermore, the absence of ACh induced a reduction in body weight and in the time of wire hang test in mutant mice. These data corroborate previously results that VAChT mice are myasthenic and had impairment in neuromuscular development and function [15, 16].

Inflammatory responses are characterized by both endothelial permeability alteration and inflammatory cell recruitment. We noticed both phenomena in mutant mice in which we found increased mononuclear cells, peribronchial edema around airways and increase in the amount of total protein in BALF when compared to wild-type mice. Additionally, an increase in the number of macrophages, lymphocytes, eosinophils and neutrophils was recovered in BALF of mutant mice. Although the inflammatory response was mild, is important to note that these animals were not submitted to any stressors to induce lung inflammation. To our knowledge, these data show for the first time that VAChT reduction induces pulmonary inflammation.

We evaluated pro-inflammatory cytokines (TNF-α, IL-6, IL-4 and IL-13) and the regulatory cytokine IL-10. Mutant mice presented higher levels of TNF-α and IL-4 in lung homogenates when compared to wild-type mice, while no difference was observed in the levels of IL-6, IL-13 and IL-10 between these two groups. IL-6 is an important cytokine involved in infection and in traumas, and is secreted primary by T cells and macrophages. IL-13 as IL-4 are more involved in allergic inflammation and are increase in experimental model of asthma [33] and IL-13 is strongly associated to mucus production be epithelial cells. The increased levels of TNF-α and IL-4 could explain the inflammation and peribronchiolar edema observed in the lung of mutant mice. Plasma extravasation is one of the first characteristics of inflammation and it is associated with TNF-α an acute mediator of inflammation [42]. Mazzon et al. [43] showed that TNF-α knockout mice present a reduction in lung inflammation and in paw edema induced by carrageenan. IL-10 is a pro-inflammatory cytokine, however, other authors have not been able to show that cholinergic anti-inflammatory system acts in IL-10 [6], corroborating our results.

Several studies have previously indicated that cholinergic activity has a fundamental role in anti-inflammatory responses in different experimental models. Vagotomized mice presented an increase in inflammatory cells in peritoneal fluid after septic peritonitis, enhancing early and late inflammatory responses [40]. However, it should be noted that vagotomy will impair not only the release of ACh, but also the secretion of peptides and potential co-transmitters [44]. Hofer et al. [45] showed that inhibition of acetylcholinesterase by physostigmine reduced lethality and circulating pro-inflammatory cytokines TNF-α, IL-1β, and IL-6 as wells as down-regulated NF-kB activity in a sepsis model. Additionally, corroborating this data, Borovikova et al. [6] suggested that ACh attenuates inflammation by a direct effect in pro-inflammatory cytokines inhibition, instead of an effect in anti-inflammatory cytokines. In the lung, Kox et al. [46] showed that vagotomy enhanced pulmonary inflammation induced by LPS, however the authors did not find any effect of vagus nerve stimulation ameliorating this response. Collectively our data expand these observations showing that VAChT function, and consequent endogenous release of ACh, is involving in controlling local inflammatory response in the lung and avoiding exacerbated inflammation even on the absence of any injury. This suggests that inflammation is kept in check by cholinergic activity.

The persistence of chronic inflammation induces tissue repair [2, 34, 35]. Tissue repair has an important clinical significance in respiratory diseases as it contributes to the worsening of lung function over the years in asthmatic and COPD patients [47]. VAChT mutant mice showed increased collagen content when compared to WT mice. Chronic inflammation induced by VAChT deficiency could explain *per se* the ECM remodeling since macrophages and other cells release different types of profibrotic mediators [48]. An imbalance between MMP and TIMP has a role in the immunomodulatory mechanisms regulating ECM composition. Additionally, MMPs are involved in inflammatory cell recruitment and tissue repair [49].

We also found higher number of positive inflammatory cells to MMP-9 and TIMP-1 in airways in VAChT mutants compared to wild-type mice, with the increase in TIMP-1 more prominent than the increase in MMP-9. That is, while MMP-9, which degrades ECM, increased less that twice in VAChT KD^HOM^ group related to baseline values (WT group), TIMP-1 increased approximately more than three fold, consistent with the dynamic turnover of ECM components that can occurs in inflammatory disease [38]. These results suggest that the chronic inflammatory unbalance due to cholinergic dysfunction may drive remodeling in the lungs.

Although no difference in basal lung function was observed, VAChT KD mice presented an increased in maximal response of respiratory system resistance to a bronchoconstrictor agonist. Airway smooth muscle contraction is an important determinant of lung mechanical alterations in a presence of a bronchoconstrictor. However, it should be noted that the presence of inflammation, edema and airway remodeling can also increase Rrs responses by reducing airway lumen diameter. In this context, mutant animals presented increased airway inflammation, edema and remodeling which could explain the hyperresponsiveness observed. Another possibility is that changes in muscarinic receptors that could appear in mutant mice as a compensatory mechanism due to ACh deficiency. In this regard, we evaluated M2 protein in lung and we did not found any difference between the groups. Verbout et al. [50] showed that atropine treatment increased antigen challenge-induced airway hyperreactivity and this effect was dependent on inflammation, particularly eosinophils, corroborating in part our findings.

Considering the mechanisms involved in VAChT deficiency-induced pulmonary inflammation, recently it has been suggested that stimulation of cholinergic receptors suppresses acute lung inflammation in mice, probably activating α7nAChR [4]. Some authors identified the presence of α7nAChR in the surface of immune cells which, following activation, regulates inflammation in a cholinergic dependent manner [9]. Activation of α7nAChR and triggers the JAK-2-STAT-3 pathway seems to inhibit the nuclear translocation of NF-kB [8]. We then evaluated the lung expression of p65-NF-kB as well as the number of cells that express p65-NF-kB and we found that mutant mice presented an increase in p65-NF-kB expression in lung, suggesting an activation of this pathway.

JAK-2 expression in lung of VAChT mutant mice was reduced. Janus kinases (JAKs) are regulators of signaling through cytokine receptors and the role of JAK2-STAT3 pathway in inflammation was not completely elucidated. Particularly in lung, this pathway was poorly investigated. The inhibition of JAK-2 prevents LPS-induced STAT-3 tyrosine phosphorylation [51]. In turn, the activation of STAT-3, induces the increase in SOCS-3 which is related to an anti-inflammatory action since it counteracts macrophage (M1) proinflammatory phenotypes [52] and inhibits NF-kB translocation [53]. Interestingly, we found that total and phosphorylate STAT-3 and SOCS-3 expression was not different between WT and mutant mice, although a tendency in reduction of STAT-3 was observed. These results suggest that in the lung, VAChT reduction and inhibition of cholinergic tone induced a reduction in JAK-2 activation that could prevent the activation of STAT-3, which in turn did not stimulate an increase in SOCS-3 in order to counteract lung inflammation. Corroborating part of this idea, de Jonge et al. [37] showed that vagal nerve stimulation improved inflammation in a model of intestinal surgery by activating STAT3 in intestinal macrophages and the authors concluded that the anti-inflammatory effects of vagal stimulation is mediated by JAK2-STAT3 activation thorough α7nAChR stimulation.

Considering that both neuronal (parasympathetic nerves) and non-neuronal (epithelial and immune cells) sources of ACh co-exist in lung, the limitation of the present study performed *in vivo* is the difficult to distinguish the effect of each pathway. In lung, is not totally clear if the source of ACh by non-neuronal cells is dependent of VAChT. Other mechanism of ACh secretion in epithelial cells has been suggested mainly related to ACh release directly from the cytoplasm [26, 54]. Is important to note that we evaluated CHT1, AChE, M2 and α7nAChR and none of them is altered in mutant mice. In addition, Lips et al. [19] showed immunoreactivity to VAChT in airways and also showed a reduction of the cholinergic machinery, including VAChT, in a model of acute airway inflammation. Recently, in an elegant review, Yang et al. [55] pointed out the importance of pulmonary parasympathetic inflammatory reflex as a regulator of lung inflammation and immunity and suggest that neuronal ACh is important to induce the release of non-neuronal ACh by immune cells in order to produce anti-inflammatory effects. On the basis of the current state of knowledge, there is no unequivocal evidence that VAChT deficiency both in the nervous system and in the lung contribute to the control of lung inflammation.

In conclusion, we showed for the first time that long-term VAChT deficiency induced airway hyperresponsiveness, inflammation and remodeling in a murine model of allergic airway inflammation. The pro-inflammatory *milieu* observed was associated to increased p65-NF-kB and inhibition of JAK-2 expression. Importantly, these data suggest that intact cholinergic tone is an important mechanism to keep in check exacerbated inflammatory responses in order to maintain the lung homeostasis. Because the lung are constantly exposed to compounds from the environmental that can induce inflammatory responses, this regulatory mechanism may be relevant to avoid aberrant reactions in response to irrelevant stimuli.
